# Prevalence, risk factors and correlation with cardiac involvement of carpal tunnel syndrome in amyloidosis

**DOI:** 10.1186/1750-1172-10-S1-P32

**Published:** 2015-11-02

**Authors:** Agnese Milandri, Simone Longhi, Christian Gagliardi, Mario Cinelli, Serena Foffi, Ilaria Bartolomei, Fabrizio Salvi, Claudio Rapezzi

**Affiliations:** 1 Diagnostic and Specialty Medicine – DIMES, Alma Mater Studiorum, University of Bologna, Cardiology, 40138, Bologna, Italy; 2 Bellaria Hospital, Neurology, 40100, Bologna, Italy

## Background

Carpal tunnel syndrome (CTS) is one of the most common clinical manifestations of TTR-related amyloidosis, both hereditary (ATTR), and wild type (senile systemic amyloidosis, SSA) and often precedes cardiac symptoms. The exact prevalence of CTS in light-chain amyloidosis (AL), ATTR and SSA is not known. We therefore aimed to establish prevalence, risk factors and possible association with cardiac involvement in patients with TTR-related and AL amyloidosis.

## Methods

We retrospectively analyzed clinical and instrumental (ECG and echocardographic) findings of 260 patients with TTR-related, and 175 with AL amyloidosis evaluated at our Centre between 1990 and September 2013.

## Results

Prevalence was 35% in TTR-related amyloidosis (35% in ATTR and 32% in SSA) and 8% in patients with AL (p< 0.001). Among TTR patients, CTS was more frequently associated with cardiac involvement (76% vs. 42%; p<0.0001) as reflected by the presence of pathological ECG and echocardiogram. Moreover, CTS manifested 9 years before the onset of cardiac symptoms. Among patients with cardiomyopathy with/without CTS there were no significant clinical/instrumental differences. At univariate analysis male gender and genotype were not associated with CTS.

## Conclusion

CTS is specifically associated with TTR-related (but not AL) amyloidosis independently from patient gender. In TTR-related amyloidosis, CTS is more frequently associated with cardiac involvement, even though patients with cardiomyopathy with/without CTS have a comparable clinical/instrumental profile. CTS precedes cardiac symptoms onset by 9 years and this awareness is important for an early diagnosis of amyloidotic cardiomyopathy.

